# Imaging joy with generalized slice dithered enhanced resolution and SWAT reconstruction: 3T high spatial–temporal resolution fMRI

**DOI:** 10.3389/fnimg.2025.1537440

**Published:** 2025-06-03

**Authors:** Jennifer D. Townsend, Angela Martina Muller, Zanib Naeem, Alexander Beckett, Bhavesh Kalisetti, Reza Abbasi-Asl, Congyu Liao, An Thanh Vu

**Affiliations:** ^1^Department of Radiology and Biomedical Imaging, University of California, San Francisco, San Francisco, CA, United States; ^2^VA Advanced Imaging Research Center, San Francisco Veteran Affairs Health Care System, San Francisco, CA, United States; ^3^Northern California Institute for Research and Education, San Francisco, CA, United States; ^4^Department of Neurology, University of California, San Francisco, San Francisco, CA, United States; ^5^UCSF Weill Institute for Neurosciences, San Francisco, CA, United States; ^6^School of Medicine, National University of Natural Medicine, Portland, OR, United States; ^7^Advanced MRI Technologies, Sebastopol, CA, United States; ^8^Helen Wills Neuroscience Institute, University of California, Berkeley, Berkeley, CA, United States; ^9^Department of Bioengineering and Therapeutic Sciences, University of California, San Francisco, San Francisco, CA, United States; ^10^Radiological Sciences Laboratory, Stanford University, Stanford, CA, United States

**Keywords:** joy, emotion, machine learning, gSLIDER, mesoscale, fMRI, SWAT

## Abstract

To facilitate high spatial–temporal resolution fMRI (≦1mm^3^) at more broadly available field strengths (3T) and to better understand the neural underpinnings of joy, we used SE-based generalized Slice Dithered Enhanced Resolution (gSLIDER). This sequence increases SNR efficiency utilizing sub-voxel shifts along the slice direction. To improve the effective temporal resolution of gSLIDER, we utilized the temporal information within individual gSLIDER RF encodings to develop gSLIDER with Sliding Window Accelerated Temporal resolution (gSLIDER-SWAT). We first validated gSLIDER-SWAT using a classic hemifield checkerboard paradigm, demonstrating robust activation in primary visual cortex even with stimulus frequency increased to the Nyquist frequency of gSLIDER (i.e., TR = block duration). gSLIDER provided ~2× gain in tSNR over traditional SE-EPI. GLM and ICA results suggest improved signal detection with gSLIDER-SWAT’s nominal 5-fold higher temporal resolution that was not seen with simple temporal interpolation. Next, we applied gSLIDER-SWAT to investigate the neural networks underlying joy using naturalistic video stimuli. Regions significantly activated during joy included the left amygdala, specifically the basolateral subnuclei, and rostral anterior cingulate, both part of the salience network; the hippocampus, involved in memory; the striatum, part of the reward circuit; prefrontal cortex, part of the executive network and involved in emotion processing and regulation [bilateral mPFC/BA10/11, left MFG (BA46)]; and throughout visual cortex. This proof of concept study demonstrates the feasibility of measuring the networks underlying joy at high resolutions at 3T with gSLIDER-SWAT, and highlights the importance of continued innovation of imaging techniques beyond the limits of standard GE fMRI.

## Introduction

Whole brain functional magnetic resonance imaging (fMRI) at high spatial–temporal resolution (≦1mm^3^) is invaluable for studying smaller subcortical structures like the amygdala and the brain’s laminar and columnar functional organization (Yacoub et al., 2007, 2008; Kashyap et al., 2018). However, given that the tSNR is generally insufficient for sub-millimeter fMRI at 3T, these studies have been limited to ultra-high field strengths (≥7T) that are prohibitively expensive in most parts of the world. Furthermore, standard fMRI technology relies on GE-EPI that, especially at higher field strengths and resolutions, suffers from large vein bias and susceptibility-induced signal dropout (Engel et al., 1997; Norris et al., 2002; Parkes et al., 2005) in brain regions essential in emotion processing (Townsend and Altshuler, 2012). This limits our ability to acquire true, whole brain, high resolution fMRI. Even with advanced acceleration techniques, scanning the entire brain at high resolution currently requires unacceptably long repetition times (TR > > 3 s).

Originally developed for sub-millimeter diffusion MRI at 3T (Setsompop et al., 2018), we found that spin-echo based generalized Slice Dithered Enhanced Resolution (gSLIDER) is a promising technique for enabling high-resolution fMRI at 3T (Beckett et al., 2021; Torrisi et al., 2023). It reduces both large vein bias and susceptibility-induced signal dropout relative to GE-EPI, and more than doubles SNR efficiency relative to traditional spin-echo based fMRI (Vu et al., 2018; Setsompop et al., 2018). However, for spins to properly relax between gSLIDER shots, the effective repetition time (TR) of gSLIDER is long (~18 s), which is incompatible with most fMRI paradigms and leaves the sequence vulnerable to blurring from head motion. To address the inherently low temporal resolution of gSLIDER fMRI, we developed a novel reconstruction method: Sliding Window Accelerated Temporal resolution (SWAT) that provides up to a five-fold increase in gSLIDER temporal resolution (TR ~ 3.5 s).

After validating gSLIDER-SWAT for high spatial–temporal resolution fMRI with a basic visual task, we applied it to investigate the neural networks underlying the emotion joy. Frontotemporal-limbic regions may benefit particularly from the enhanced spatial resolution and improved signal quality of this SE-based technique, as these regions are prone to susceptibility-induced signal dropout and geometric distortions with standard GE techniques due to their proximity to air-tissue interfaces (Ojemann et al., 1997; Beckett et al., 2020; Townsend et al., 2010, 2019; Yacoub et al., 2008). This integrated approach enables us to validate the gSLIDER-SWAT technique and demonstrate its application to address important neuroscientific questions that have been limited by conventional imaging approaches.

Emotion processing involves complex neural networks that detect, evaluate and regulate affective and visceral responses to environmental stimuli (Barrett et al., 2007). Emotions are characterized by specific patterns of neural and autonomic activation, coordinated through reciprocal connections between corticolimbic structures and systems governing physiological arousal. fMRI emotion studies show significant activation in the amygdala, insula, anterior cingulate cortex (ACC), medial prefrontal cortex (mPFC) and ventrolateral prefrontal cortex (vlPFC) (Phillips et al., 2003a). The amygdala is part of the salience network and is involved in emotion detection and processing, while medial and lateral regions of the PFC are critical for emotion modulation and regulation (Ochsner et al., 2012; Townsend et al., 2013). Recent work further describes the role of amygdala subregions in different emotions (Labuschagne et al., 2024), highlighting the importance of ultra-high resolution in this region.

Positive emotions also activate regions associated with reward processing (Schultz et al., 1997), including the ventral striatum, nucleus accumbens, ACC and orbitofrontal cortex (Wager et al., 2015; Suardi et al., 2016; Yang et al., 2020). Recent studies show that positive emotions encompass emotions with discrete neural representation (ex: joy vs. awe vs. sexual desire) (Cowen and Keltner, 2017; Saarimäki et al., 2016, 2018), which has been shown across a range of stimuli (Cowen and Keltner, 2018, 2020; Cowen et al., 2019). fMRI studies examining joy in the context of music have found activation in these same reward-related regions (Koelsch and Skouras, 2014; Skouras et al., 2014). This neural evidence aligns with Buddhist contemplative traditions, which recognize joy (*muditā*) as one of four fundamental qualities of mind that can be intentionally cultivated and expanded (Davidson and Lutz, 2008; Esch, 2022). While traditional emotion research predominantly has focused on negative emotions, these ancient contemplative insights and innovative neuroscience methods motivate our investigation into the neural mechanisms underlying positive emotions, specifically joy.

Studies using naturalistic stimuli have begun to reveal the brain’s hierarchical temporal processing involving the hippocampus, attentional mechanisms, and basic and social emotional processing (Dayan et al., 2018; Jääskeläinen et al., 2021). There has only been one study to date investigating the neural underpinnings of joy using naturalistic video stimuli, despite its importance in studying emotion. A recent fMRI study decoded 27 categories of emotions (Horikawa et al., 2020). While decoding joy using standard GE sequence at 3T, they show significant cortical regions, but not subcortical regions like the amygdala. This was surprising given that the amygdala is activated in response to positive emotions in general (Bonnet et al., 2015; Townsend et al., 2017). Given the GE sequence, signal dropout in these inferior regions may have contributed to the lack of significant activity; thus, we investigated whether we see significant limbic activity with these same naturalistic joy stimuli using gSLIDER.

We hypothesize that gSLIDER-SWAT will provide a gain in temporal resolution by recapturing high frequency information and will increase the tSNR relative to traditional SE-EPI, resulting in improved detection of functional networks at high resolutions at 3T.

## Methods

The study protocol was approved by the institutional review board at the University of California, San Francisco; each participant gave written informed consent before initiating the study. Due to scanner availability at the time, fMRI data was acquired from 2 healthy volunteers initially on a Siemens 3T Prisma using a 64ch head/neck coil, and then from 6 volunteers (age= 42 ± 13; 1F/4M) on a Siemens 3T Skyra using a 32ch head coil. Data from one volunteer was excluded due to excessive motion.

### Sequence parameters

**Figure 1:**
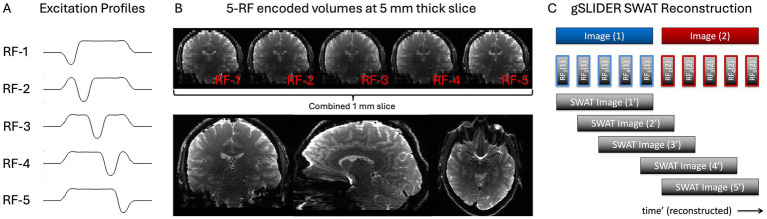
gSLIDER factor 5 acquisition uses five thin-slab volumes that are five times the thickness of the final slice resolution (e.g., 5 mm slab - > 1 mm slices). Each of the five thin-slab volumes are acquired with different slice phase–dither encoding **(A)** and then combined to create the high-resolution image **(B)**. SWAT gSLIDER reconstruction for 5× gain in temporal resolution **(C).** Blue Image (1) and Red Image (2) represent two adjacent timepoints of the original gSLIDER method each made up of five of their own dithered RF excitations.

FOV = 220 × 220 × 130 mm^3^; resolution = 1 × 1 × 1 mm^3^, PF = 6/8; GRAPPA 3; TE = 69 ms; TR = 18 s (3.6 s per dithered volume). To achieve this resolution for gSLIDER factor 5, 26 thin-slabs (5 mm thick) were acquired, each acquired 5× with a different slice phase (Figures 1A,B). 1mm^3^ iso SE-EPI tSNR scans were acquired for comparison using matched to the gSLIDER scan parameters (TR = 18 s). A short GE-EPI scan was run for image quality comparison using the same imaging parameters but with TE = 35 ms.

### gSLIDER reconstruction SWAT

We used custom MATLAB reconstruction code using magnitude, phase and B1 maps. The thin, 1 mm slices are reconstructed from the five sequentially acquired thin-slabs using standard linear regression with Tikhonov regularization: *z* = *(A^T^A + λI)^−1^ A^T^b* = *A_inv_b*, where *A* is the forward transformation matrix containing the spatial RF-encoding information, *λ* is the regularization parameter (set to 0.1), *b* is the concatenation of the acquired RF-encoded slab data, and *z* is the high-resolution reconstruction. Bloch simulated slab profiles were used to create the forward model (A) which allows the reconstruction to account for the small cross-talk/coupling between adjacent slabs (Setsompop et al., 2018).

For illustrative purposes, the matrix *A* for 5×-gSLIDER can be roughly approximated as: [−1 1 1 1 1; 1 −1 1 1 1; 1 1 −1 1 1; 1 1 1 −1 1; 1 1 1 1 −1]. This presumes that the underlying signal is stationary. While true for anatomical imaging like diffusion MRI, for fMRI imaging, each of the sequentially acquired slabs contains useful temporal information amenable to a sliding window reconstruction (Figure 1C), where for example the next TR could be reconstructed using the following shifted A matrix: [1 −1 1 1 1; 1 1 −1 1 1; 1 1 1 −1 1; 1 1 1 1 −1; −1 1 1 1 1]. We call this “view-sharing” approach Sliding Window Accelerated Temporal resolution (SWAT) and evaluate the expected 5× gain in temporal resolution and resultant statistical power. The sliding window reconstruction in gSlider-SWAT acts as a temporal frequency-domain filtered upsampling that unaliases and recovers, albeit at an attenuated level, higher-frequency hemodynamic components. This is done by exploiting the unique spatial–temporal information from the five RF excitation profiles per slab, which should enable detection of BOLD activity changes not visible with the original gSLIDER method.

### Task 1 Hemifield

**Figure 2:**
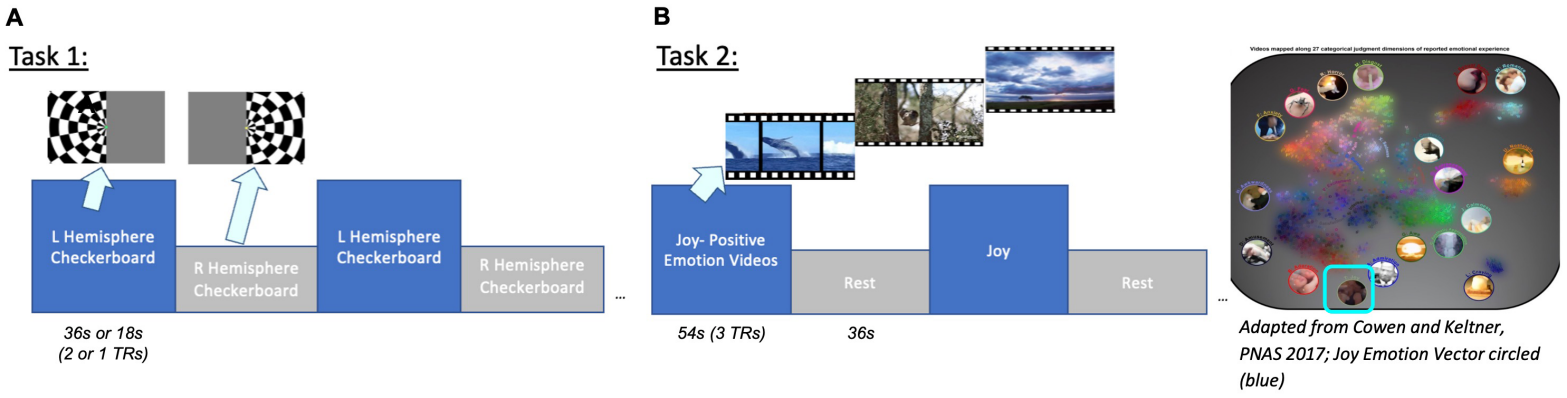
Task designs. Task 1: Visual hemifield localizer stimulus (36 s or 18 s blocks corresponding to 2 or 1 TRs). Task 2: Positive Emotion Joy naturalistic video stimuli (from Cowen and Keltner, 2017). Videos were clustered by emotion vector to group clips with the highest average cosine similarity. Groups of clips with the highest joy category and valence ratings were presented.

Initial evaluation of the gSLIDER-SWAT sequence was performed using a visual hemifield localizer stimulus consisting of alternating left vs. right visual hemifield flickering checkerboards: 36 s per hemifield block, T = 72 s, 9 repeats per run; 2 runs per subject (Figure 2). Subsequent testing increased the stimulation frequency to the Nyquist frequency of gSLIDER (18 s per hemifield block, T = 36 s, 18 repeats per run). Stimuli were presented using PsychoPy3 and an Avotec SV-6060 Projector. Throughout the hemifield stimulus, volunteers were tasked with focusing on a fixation dot at the center of the screen and to press a button each time the dot turned yellow.

### Task 2 Joy

Positive Emotion video stimuli (from Cowen and Keltner, 2017; clustered by emotion vector to group clips with the highest average cosine similarity; presented groups of clips with highest valence, arousal and joy category ratings; identical timing as task 1, alternating videos on and rest). The problem of clustering together video clips is defined as follows: given a collection of video clips, each with a duration and emotions vector, group the clips such that each group contains clips with similar emotions vectors and the total duration of each group is ~60s. To group the video clips, we use a greedy algorithm, an approach that makes the locally optimal choice at each step. To build a group, we greedily add the video clip with the highest average cosine similarity to the group that also satisfies the group’s maximum duration constraint with some slack. This process is repeated until the total duration of the group reaches approximately 1 min, after which a new group is created. Videos with the highest joy vectors and highest valence (example clips: Corgi puppies running in grass, babies playing with toys, a blind man seeing for the first time; ave. valence = 7.13; arousal = 5.93; joy rating = 0.25) were selected for video clips, with 54 s on alternating with 36 s rest (cross-hair fixation) for a total of 6 cycles = 540 s/run.

### Ljung-Box test

To validate use of FILM to account for the temporal autocorrelation in the fMRI time series, we performed Ljung-Box tests on the GLM residuals for gSLIDER (GS) and gSLIDER-SWAT. The Ljung-Box tests whether autocorrelations of the residuals are significantly different from zero for a given number of lags (Ljung and Box, 1978). Given the hemodynamic response function is on the order of 30–40s, we selected a 36 s window; with TR_GS = 18 s and TR_SWAT = 3.6 s, we tested lags GS nlag = 2, SWAT nlag = 10 capture both short- and long-range temporal dependencies (Yue et al., 2024).

### fMRI analysis Hemifield

FMRI data processing was performed using FEAT (FMRI Expert Analysis Tool) v6.00; FSL (FMRIB’s Software Library, www.fmrib.ox.ac.uk/fsl). Independent Component Analysis (ICA) was performed on gSLIDER with and without SWAT reconstructions using FSL’s MELODIC with signal and noise components manually identified. Pre-processed data were whitened and decomposed into sets of vectors to describe signal variation across temporal and spatial domains (Beckmann and Smith, 2004). GLM analysis was performed on the fast 18 s block stimuli data using FSL’s FEAT which included pre-whitening using FILM (Woolrich et al., 2001).

As a control comparison to ensure that the observed benefits of SWAT reconstruction are not artificially inflated due merely to the presence of additional time points (i.e., up-sampled interpolation), we also performed simple 5-fold interpolation of the gSLIDER time series utilizing MATLAB’s interpolation function (which inserts zeros into the original signal and then applies a lowpass interpolation filter at half the Nyquist frequency to the expanded sequence). This implementation allows the original data to pass through unchanged, without adding any additional temporal information, and interpolates to minimize the mean-square error between the interpolated points and their ideal values.

### fMRI Analysis Joy

GLM analysis preprocessing included: 2 mm smoothing, high pass filter (90s), FILM, 6 motion parameters regressors, and registration to MNI 2 mm atlas. For random effects group analyses, n = 6 runs were included. Z (Gaussianised T/F) statistical images were thresholded using clusters determined by Z > 1.7 and a (corrected) cluster significance threshold of *p* = 0.05 (Worsley, 2001), unmasked.

## Results

**Figure 3:**
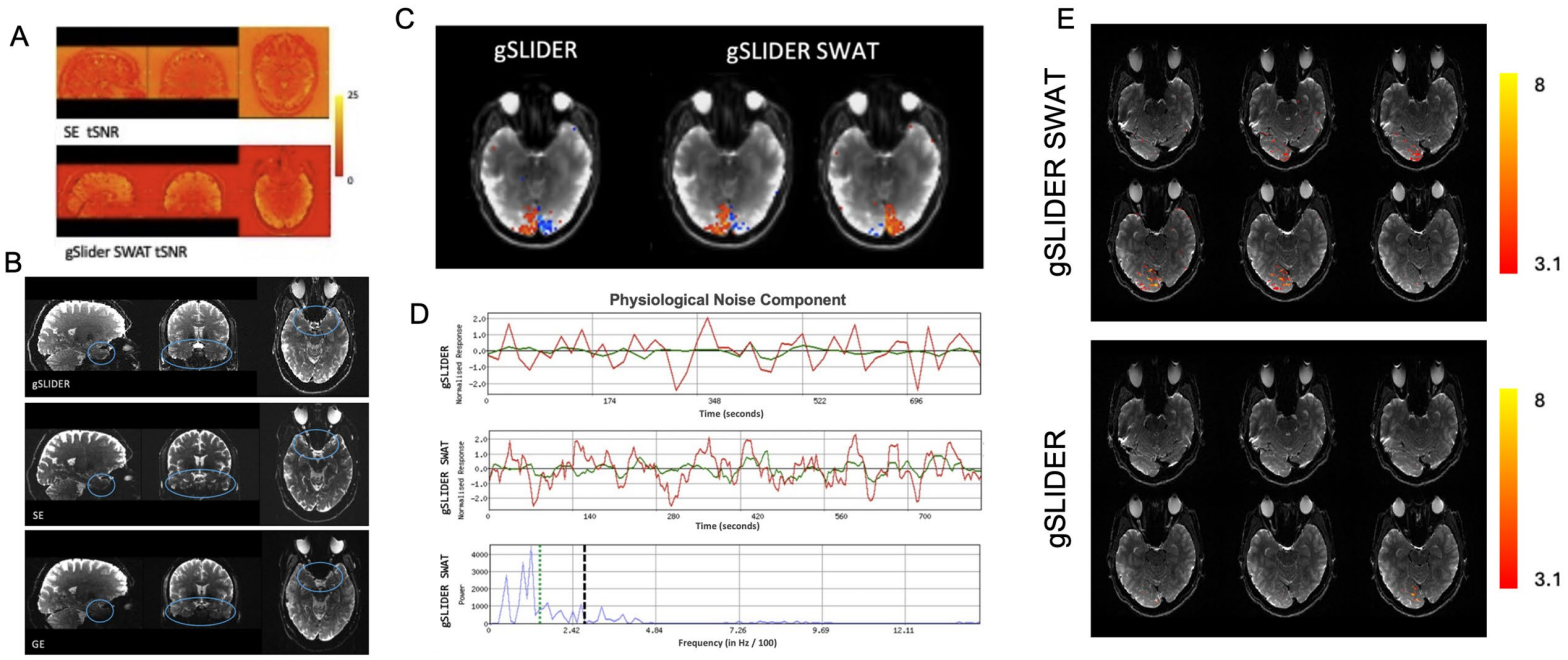
Advantages of gSLIDER SWAT. **(A)** Improved (×2) tSNR relative to SE-EPI. **(B)** Improved signal in inferior frontal and temporal regions compared to traditional GE. **(C)** Using FSL’s MELODIC analysis of visual hemifield task data, gSLIDER SWAT resolves individual hemispheric activations into two separate ICs, while gSLIDER without SWAT does not. **(D)** Time courses of a physiological noise component are shown without SWAT (top) and with SWAT (middle). The power spectra of the gSLIDER SWAT time series (bottom) clearly shows power above the Nyquist frequency of gSLIDER without SWAT (vertical dashed black line). For reference, the hemifield task frequency is indicated by the vertical dotted green line. **(E)** BOLD activations (Z > 3.1) for hemifield localizer task with stimulus frequency set to the Nyquist frequency of gSLIDER (TR = block duration = 18 s) for gSLIDER-SWAT (top) and gSLIDER (bottom).

As expected, gSLIDER showed significant tSNR gains (~2×) over traditional SE (Figure 3A), along with improved signal coverage in regions of signal dropout, such as inferior frontal and temporal regions, compared to traditional GE-EPI (Figure 3B).

### Ljung-Box

This test is used to assess temporal autocorrelation, and gSLIDER demonstrated no significant autocorrelations with or without FILM pre-whitening (all *p* > 0.29). In contrast, SWAT exhibited significant autocorrelations without pre-whitening (all *p* < 0.001), which were successfully mitigated with FILM (all *p* > 0.3). Consequently, all subsequent analyses incorporate FILM pre-whitening.

### Hemifield

Figure 3 demonstrates the advantage of gaining high frequency information with the higher temporal sampling provided by gSLIDER-SWAT. With the 5-fold increase in temporal sampling frequency with SWAT, independent components corresponding to individual visual hemifield activation patterns are resolved, while the original reconstructed 18 s temporal resolution gSLIDER data is only able to resolve merged visual activity (Figure 3C; Supplementary Figure S1). From the gSLIDER-SWAT data, MELODIC detected ~3× more independent components compared to both original gSLIDER and to the 5-fold simple interpolation of the gSLIDER data (i.e., as a control). Figure 3D shows the time courses of a physiological noise component with and without SWAT. The power spectra of the gSLIDER-SWAT time series (bottom) clearly shows power above the Nyquist frequency of gSLIDER without SWAT (vertical dashed black line). For reference, the 36 s block hemifield task frequency is indicated by the vertical dotted green line. As expected, simple interpolation of gSLIDER data did not introduce power above the Nyquist frequency (Supplementary Figure S1A). Importantly, when the stimulus frequency was increased to the Nyquist frequency of gSLIDER (TR = block duration = 18 s), gSLIDER-SWAT was still able to detect robust activity throughout visual cortex while original gSLIDER was not (Figure 3E).

### Joy

**Figure 4:**
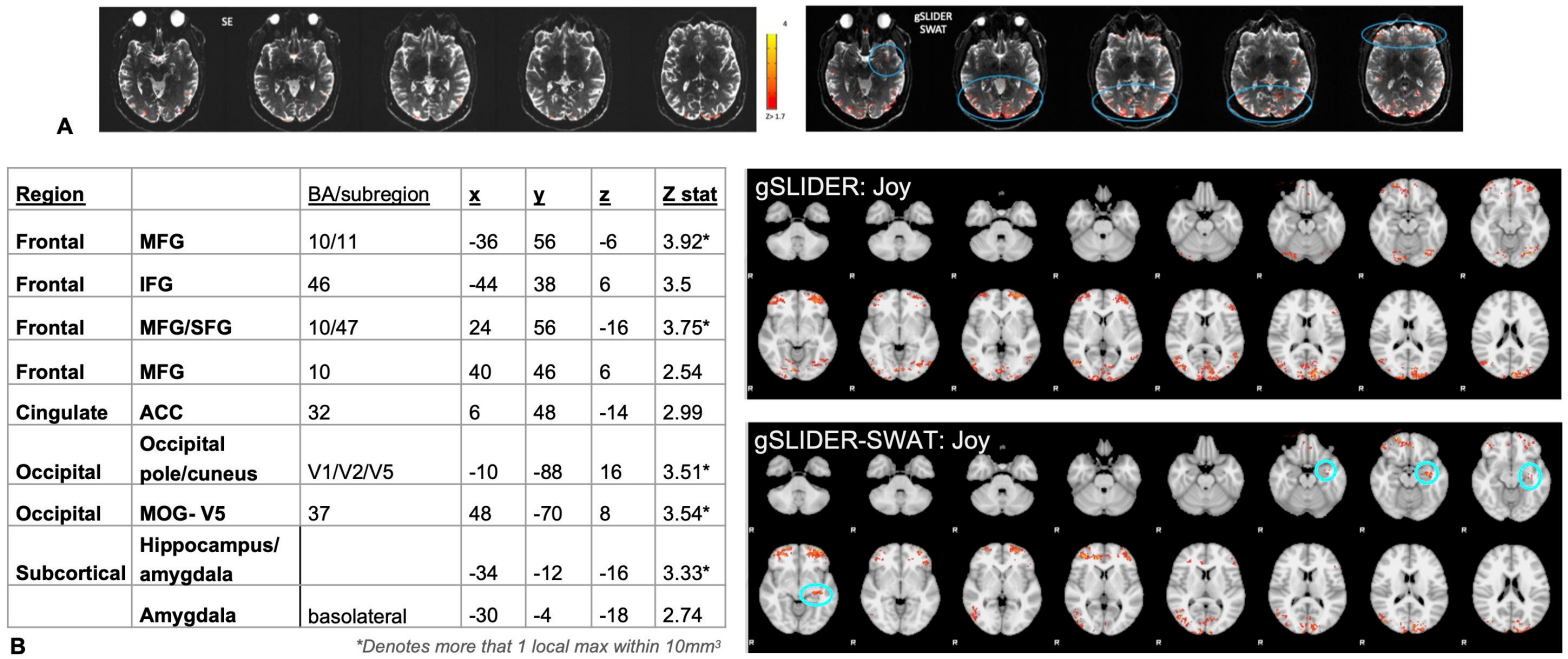
Joy. We first demonstrate improved CNR detection of the neural substrates of Joy - Fixation with gSLIDER-SWAT (**A**, circled) compared to SE. Random effects analyses (**B**. bottom table/panels, n=6 runs) reveal significant activation in occipital and frontal regions using gSLIDER, and with gSLIDER-SWAT additionally in left hippocampus/extended amygdala-basolateral subregion (circled) (3T 1mm3; Z>1.7 cluster corrected, *p*<0.05, no masking).

Initial GLM analysis showed less CNR with SE 1mm^3^ compared to gSLIDER-SWAT during the Joy task, with SE showing minimal activation restricted to visual cortex (Figure 4A). Unlike with SE, gSLIDER-SWAT showed significant activation in the extended amygdala and medial prefrontal and orbitofrontal cortex, and throughout visual cortex. Next, group random effects analyses revealed significant activation in frontal and occipital regions with both gSLIDER and SWAT: bilateral frontal (superior/middle frontal gyri, BA10/11, including rostral PFC; anterior cingulate BA32); and bilateral occipital (primary visual, middle occipital gyri including V4 and V5 and temporo-parieto-occipital junction). With SWAT, additional significant activation was seen in the left extended amygdala/hippocampus (basolateral amygdalar nucleus; Z = 3.33), with no differences reaching significance with direct comparison. For point of comparison between the reconstructions, the ACC (BA32) was significant at Z = 2.13 with GS and z = 2.99 with SWAT; a 28% gain in this *a priori* region located inferiorly near susceptible regions. At the individual subject level (Z > 2), significant results were also seen in the reward network, including the right nucleus accumbens and striatum; however, these activations did not reach statistical significance at the group level due to the limited sample size.

## Discussion

This is the first high spatial–temporal resolution fMRI study at 3T using gSLIDER-SWAT, which seeks to improve coverage and detection in areas of high signal dropout near critical brain structures like the amygdala. The amygdala, a complex structure with more than a dozen nuclei, is conserved across vertebrates and situated deep and medial in the temporal lobe in primates (Kim et al., 2011). Additionally, joy has recently been discussed conceptually across species as intense, brief, and event-driven (Nelson et al., 2023). High-resolution (≤1 mm iso) is essential for investigating the functionally distinct amygdalar subnuclei, which are small, have unique neuroanatomical connectivity and serve discrete functions. Here, we found significant activation of the basolateral (BL) nucleus of amygdala during the viewing of Joy stimuli. The BL nucleus is composed of glutamatergic pyramidal neurons (~80–90%) and GABAergic neurons, and BL interneurons receive extensive sensory inputs from cortical and thalamic regions. It has been implicated in emotional learning and memory, fear conditioning and anxiety through projections to areas like the central amygdala, prefrontal cortex and hippocampus (McDonald, 2020) and reward processing through direct excitatory connections to the nucleus accumbens (Ambroggi et al., 2008).

Additionally, at the single subject level during joy, we saw significant activation in the nucleus accumbens and striatum, key regions in the dopaminergic reward system. These findings align with prior research on the mesolimbic system, providing evidence of its involvement in joy. Dopaminergic neurons from the ventral tegmental area (VTA) primarily contribute to the mesolimbic and mesocortical pathways, projecting dopamine to the NAc and PFC (Schultz et al., 1997; Young and Nusslock, 2016). The NAc serves as a hub that links reward-related behavior, integrating inputs from cortical areas involved in executive functions (PFC) with emotional and sensory information from the limbic system. This integration facilitates goal-directed behavior, motivation and learning, and has been implicated in up-regulating positive emotions (Rueschkamp et al., 2019).

The neural circuitry of emotion is critical to study as its dysfunction contributes to various neuropsychiatric conditions including mood disorders characterized by emotion dysregulation (Phillips et al., 2003b; Altshuler et al., 2005). Understanding how these neural networks operate in healthy and clinical populations can provide important insights into the neural basis of emotion processing and its disruption in psychiatric illness (Townsend and Altshuler, 2012; Njau et al., 2020). The amygdala and prefrontal cortex share bidirectional connections, and the BL amygdala has extensive reciprocal projections with the medial and orbital prefrontal regions - a circuit critical for emotional processing, learning and regulation. Studies have shown that disrupted connectivity between these regions is associated with impaired emotion regulation and mood dysregulation (Townsend et al., 2013). Advancing our understanding of emotion circuitry requires robust high-resolution neuroimaging methods that can reliably capture neural activity in regions like the orbitofrontal cortex and amygdala. We hope this study spurs further innovation in sequence development and other alternatives to traditional GE sequences for affective neuroscience fMRI.

With GE, fMRI signal acquisition in the amygdala and the orbitofrontal cortex/PFC, can be compromised due to susceptibility artifacts arising from the adjacent air-filled cavities, particularly the sphenoid sinus and petrous portion of the temporal bone. These artifacts at the tissue-air interfaces lead to signal dropout and geometric distortions in the affected regions, limiting accurate measurement and localization. By incorporating novel SWAT reconstruction into gSLIDER, we were able to achieve both the spatial resolution necessary to resolve these small brain structures and the temporal resolution required for typical fMRI.

One recent study did apply gSLIDER to fMRI (Han et al., 2020). However, in order to achieve a high temporal resolution (TR = 1.5 s), they were limited to a gSLIDER factor of 2 and 1.5mm^3^ spatial resolution. In contrast, our study utilizes a gSLIDER factor of 5 that provides a theoretical gain of 58% higher SNR efficiency, which facilitates the factor of 3.4× finer volumetric resolution and opens the door to high-resolution fMRI at 3T. Importantly, although our initial testing utilized 1mm^3^ resolution without multiband to facilitate rapid image reconstruction, optimization and evaluation of our scan protocols, we anticipate that future work will be able to utilize higher resolutions (≤0.8 mm isotropic) in conjunction with multiband acceleration so as to maintain the same gSLIDER-SWAT TR of ~3.6 s.

SWAT is an effective novel gSLIDER reconstruction method that can further enhance BOLD signal detection by reclaiming additional high frequency temporal information. The incorporation of SWAT reconstruction into gSLIDER is a significant improvement as it accelerates and adapts an originally slow, diffusion MRI technique (TR = 18 s) to one with sufficient temporal resolution for most high-resolution fMRI applications (TR = 3.6 s). We validated the benefit of this faster sampling afforded by SWAT through several approaches including using visual stimulation at the Nyquist frequency of gSLIDER (Figure 3E). As expected when sampling at the Nyquist frequency, the ability to detect BOLD activity becomes extremely sensitive to the temporal alignment of the BOLD response and the acquisition of individual slices. This explains why just a single slice of the original gSLIDER showed significant BOLD activity. Importantly, while the broad width of the sliding window results in a temporal point-spread-function that attenuates higher spatial frequencies, it does not eliminate them (Figure 3D; Supplementary Figure S1). This is consistent with the findings of high-resolution fMRI studies evaluating the impact and optimization of spatial blurring versus sampling resolution on the ability to resolve mesoscale functional organization (Yacoub et al., 2008; Vu et al., 2018).

We also confirmed that the observed improvement in statistical power afforded by SWAT was not merely due to the faster sampling rate available (e.g., via simple 5-fold interpolation) since results using simple interpolation were similar to that of the original gSLIDER (Supplementary Figures S1–S3). However, accounting for temporal autocorrelation, a known characteristic of fMRI data, in the fMRI model for gSLIDER-SWAT is essential as they may result in inflated statistics and false positives (Supplementary Figure S4). Future investigation into the impact of different autocorrelation mitigation methods on Type 1 and Type 2 errors in the context of SWAT may be of interest. In this study the LjungBox test confirmed that use of FSL’s FILM pre-whitening was effective at removing the autocorrelations introduced by SWAT.

gSlider fMRI is an evolving technology that holds promise for advancing high-resolution neuroscience research at 3T by combining the benefits of SE with enhanced tSNR. Further refinement is required to address the current limitation of slab-boundary artifacts observed every fifth slice (Figure 1B). Future incorporation of novel techniques like pseudo Partition-encoded Simultaneous Multislab (pPRISM, Chang, 2023) may help to mitigate these limitations and facilitate continued optimization of gSLIDER for fMRI.

## Conclusion

This study is the first to demonstrate the feasibility of high spatial–temporal resolution (≦1 mm3) fMRI at 3T using gSLIDER-SWAT, a novel gSLIDER reconstruction technique that shortens the effective TR to values typically used in fMRI and offers one alternative approach to traditional GE and SE sequences. When applied to investigate the neural correlates of joy using naturalistic stimuli in this pilot sample, gSLIDER-SWAT revealed activation in the salience network and emotion processing regions including the basolateral amygdala, anterior cingulate and prefrontal cortex; in visual regions; and in reward network regions like the nucleus accumbens and striatum at the individual subject level. While further optimization is needed to address the slab-boundary artifacts to make it a viable alternative, this study hopes to advance the field of high-resolution fMRI at 3T and spur additional sequence innovation. Combining gSLIDER-SWAT’s enhanced spatial and temporal resolution and reduced susceptibility artifacts may open possibilities for investigating functional organization in regions traditionally challenged by signal dropout.

## Data Availability

The raw data supporting the conclusions of this article will be made available by the authors, without undue reservation.

## Supplementary material

SUPPLEMENTARY FIGURE S1A FSL’s MELODIC results for visual hemifield task data (36s blocks) from an example subject (Subject 1) for original gSLIDER (top), 5-fold simple interpolated gSLIDER (middle), and gSLIDER-SWAT (bottom).

SUPPLEMENTARY FIGURE S1B FSL’s MELODIC results for visual hemifield task data (36s blocks) from an additional subject (Subject 2) for original gSLIDER (top), 5-fold simple interpolated gSLIDER (middle), and gSLIDER-SWAT (bottom).

SUPPLEMENTARY FIGURE S2 FSL’s FEAT GLM results for visual hemifield task data (36s blocks) from Subject 1 with versus without FILM pre-whitening for original gSLIDER (top), 5-fold simple interpolated gSLIDER (middle), and gSLIDER-SWAT (bottom).

SUPPLEMENTARY FIGURE S3 Example voxel time courses for the visual hemifield task data (36s blocks) from Subject 1 with FILM pre-whitening for original gSLIDER (top), 5-fold simple interpolated gSLIDER (middle), and gSLIDER-SWAT (bottom).

SUPPLEMENTARY FIGURE S4 FSL’s FEAT GLM results for visual hemifield task data (36s blocks) from Subject 1 comparing SE-EPI (left), gSLIDER (middle), and gSLIDER-SWAT (right).
