# Supplementary figures and images for: Imaging joy with generalized slice dithered enhanced resolution and SWAT reconstruction: 3T high spatial–temporal resolution fMRI
Source: Front Neuroimaging. 2025 Jun 3;4:1537440. doi: 10.3389/fnimg.2025.1537440 (PMC12170577; doi:10.3389/fnimg.2025.1537440)

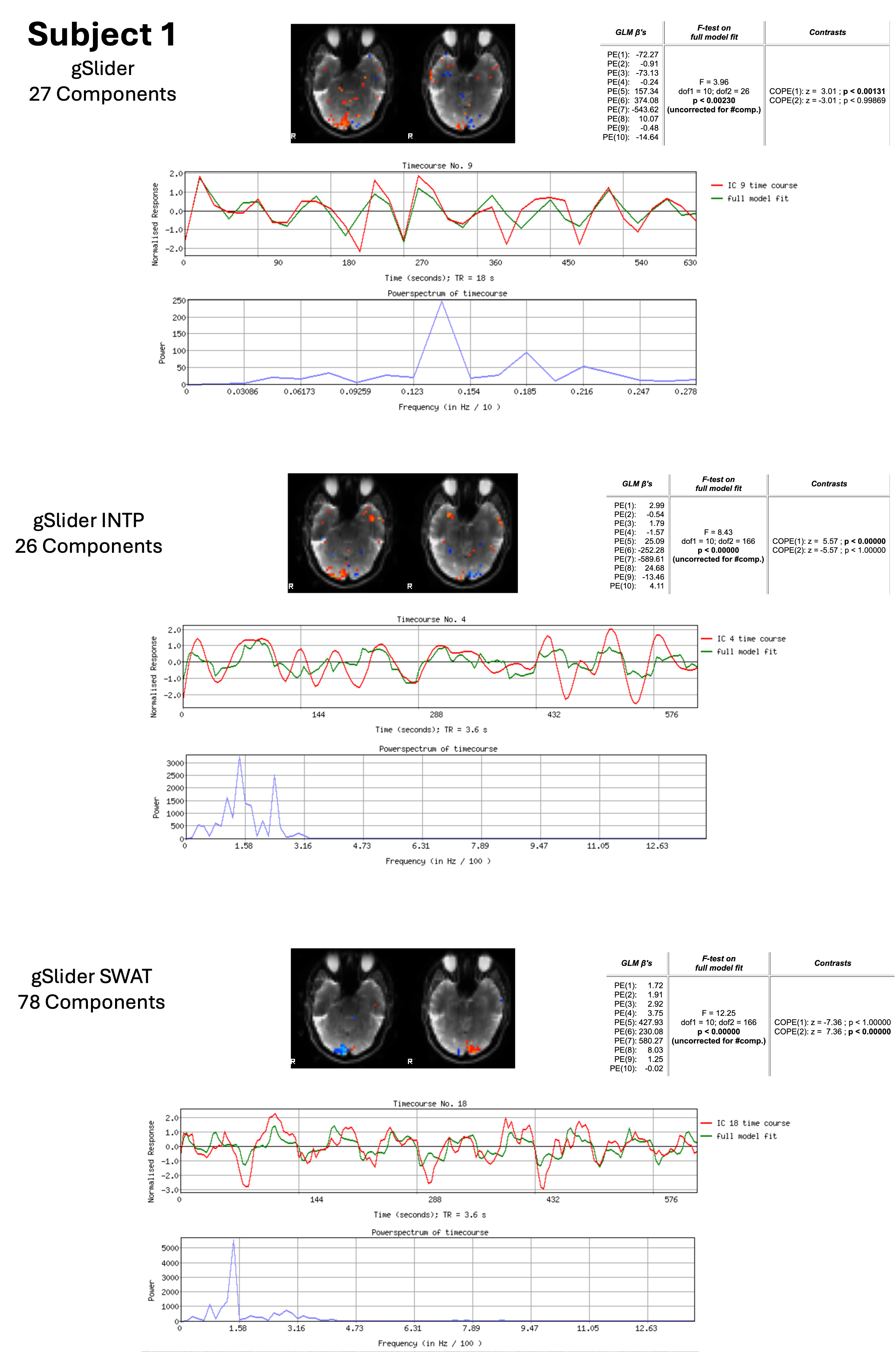

Supplement: SUPPLEMENTARY FIGURE S1A — FSL’s MELODIC results for visual hemifield task data (36s blocks) from an example subject (Subject 1) for original gSLIDER (top), 5-fold simple interpolated gSLIDER (middle), and gSLIDER-SWAT (bottom). [file Image_1.jpeg]

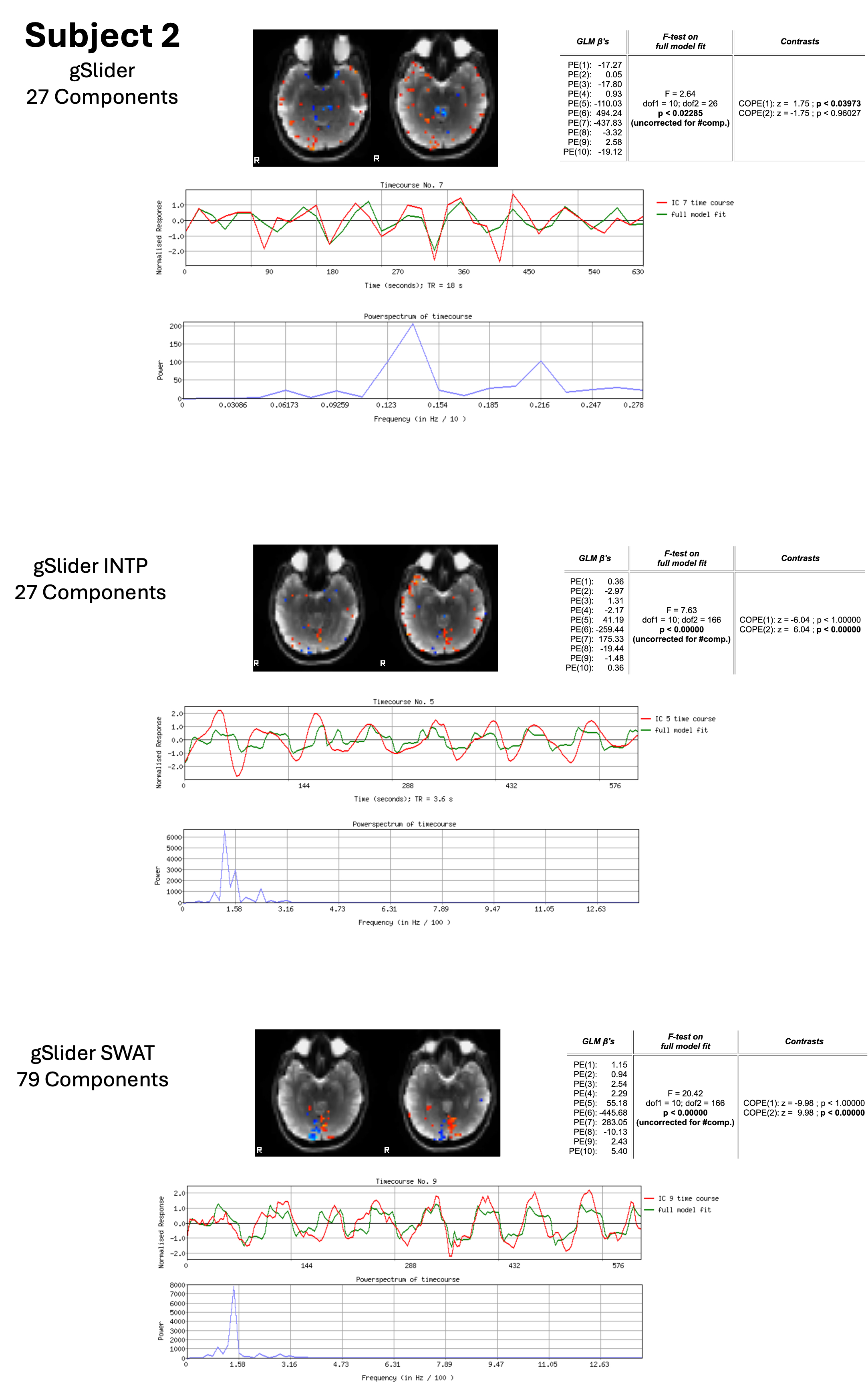

Supplement: SUPPLEMENTARY FIGURE S1B — FSL’s MELODIC results for visual hemifield task data (36s blocks) from an additional subject (Subject 2) for original gSLIDER (top), 5-fold simple interpolated gSLIDER (middle), and gSLIDER-SWAT (bottom). [file Image_2.jpeg]

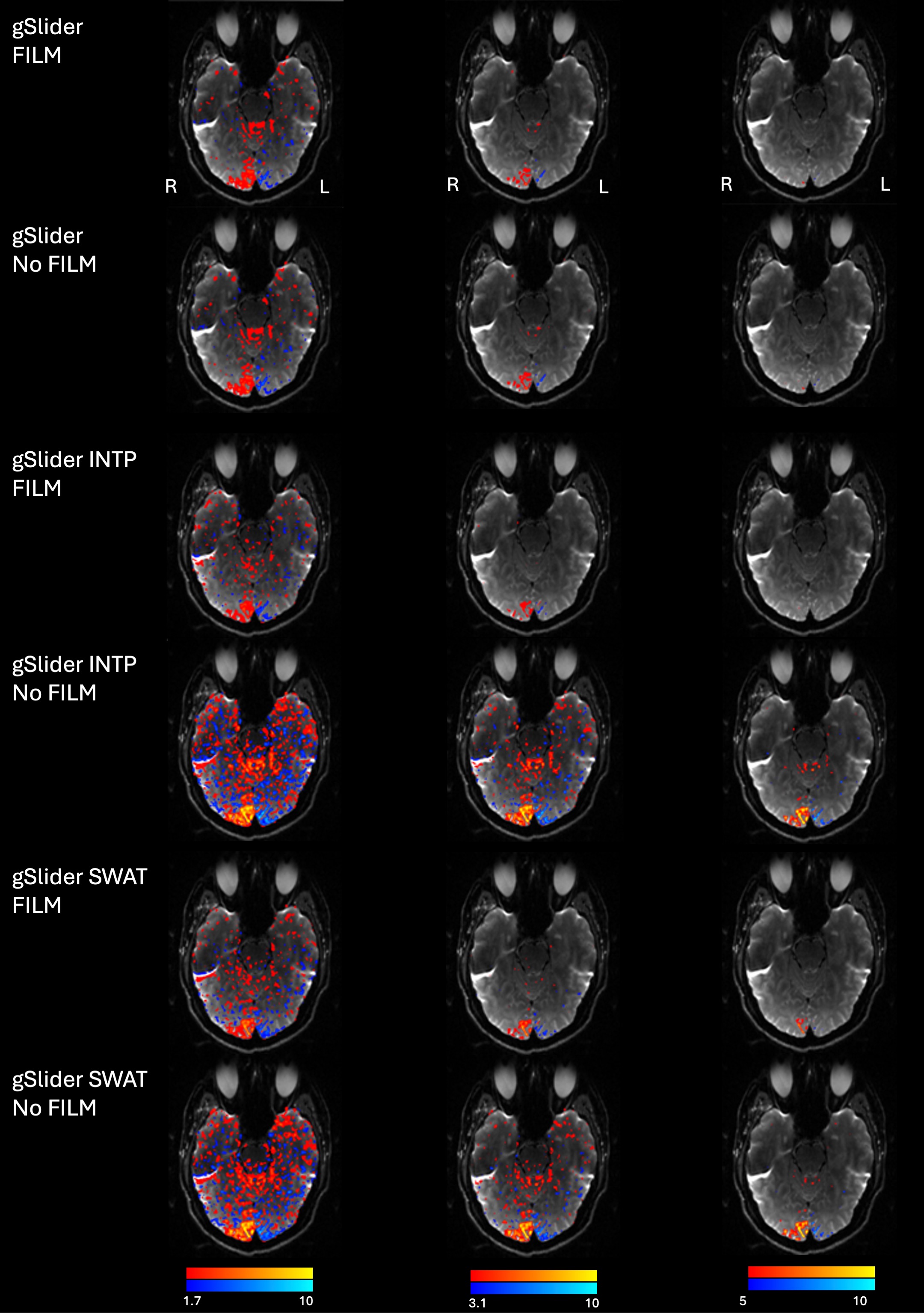

Supplement: SUPPLEMENTARY FIGURE S2 — FSL’s FEAT GLM results for visual hemifield task data (36s blocks) from Subject 1 with versus without FILM pre-whitening for original gSLIDER (top), 5-fold simple interpolated gSLIDER (middle), and gSLIDER-SWAT (bottom). [file Image_3.jpeg]

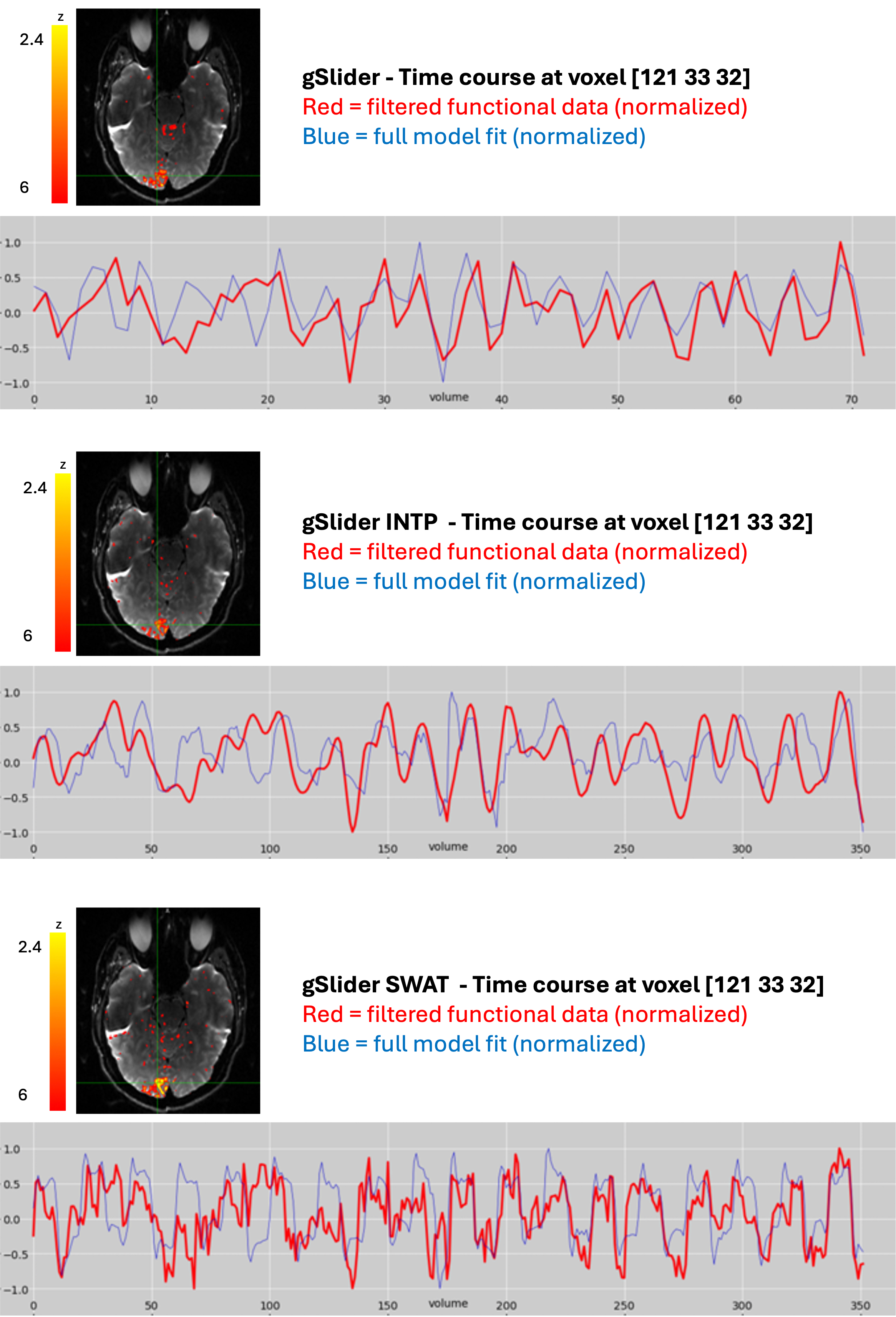

Supplement: SUPPLEMENTARY FIGURE S3 — Example voxel time courses for the visual hemifield task data (36s blocks) from Subject 1 with FILM pre-whitening for original gSLIDER (top), 5-fold simple interpolated gSLIDER (middle), and gSLIDER-SWAT (bottom). [file Image_4.jpeg]

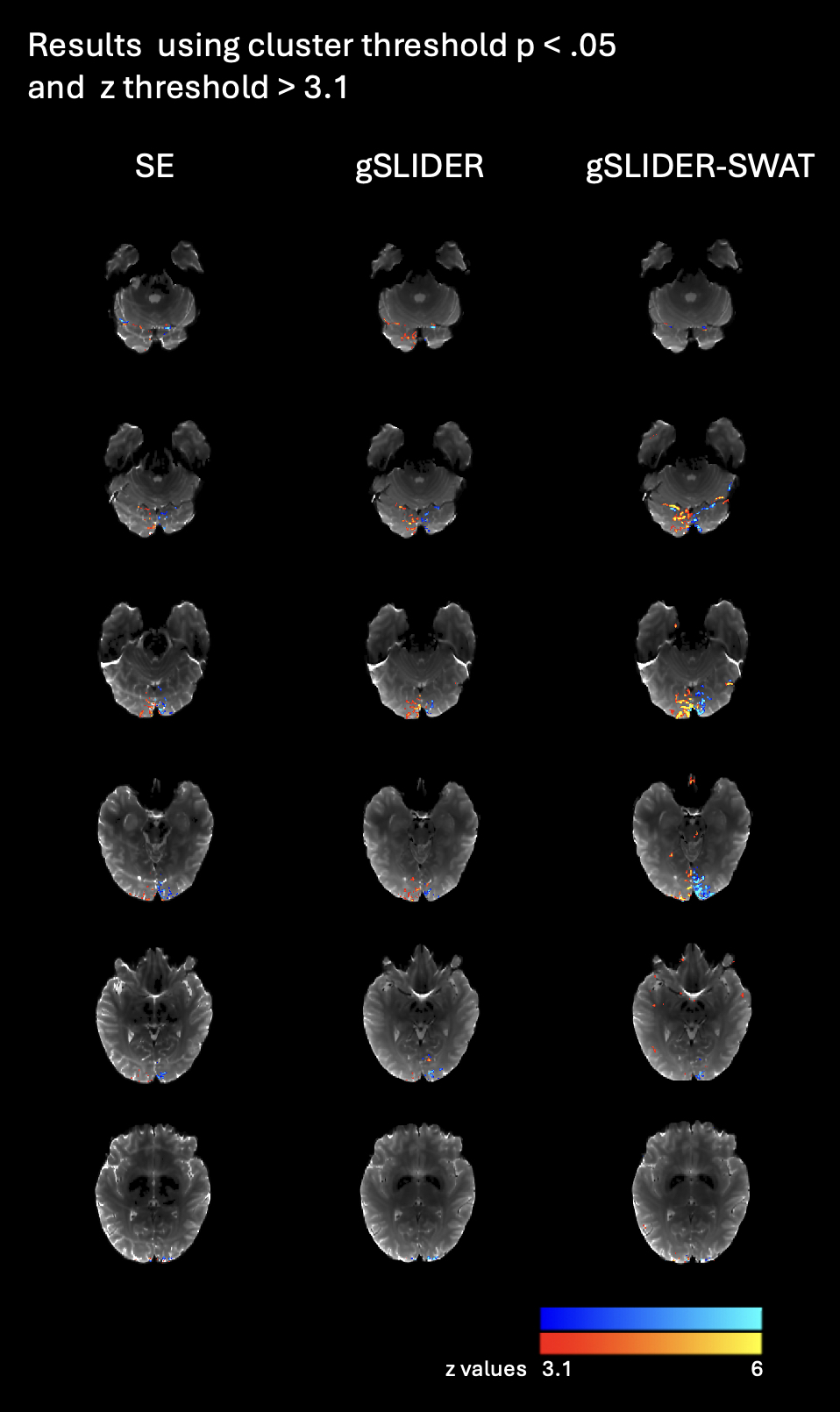

Supplement: SUPPLEMENTARY FIGURE S4 — FSL’s FEAT GLM results for visual hemifield task data (36s blocks) from Subject 1 comparing SE-EPI (left), gSLIDER (middle), and gSLIDER-SWAT (right). [file Image_5.jpeg]

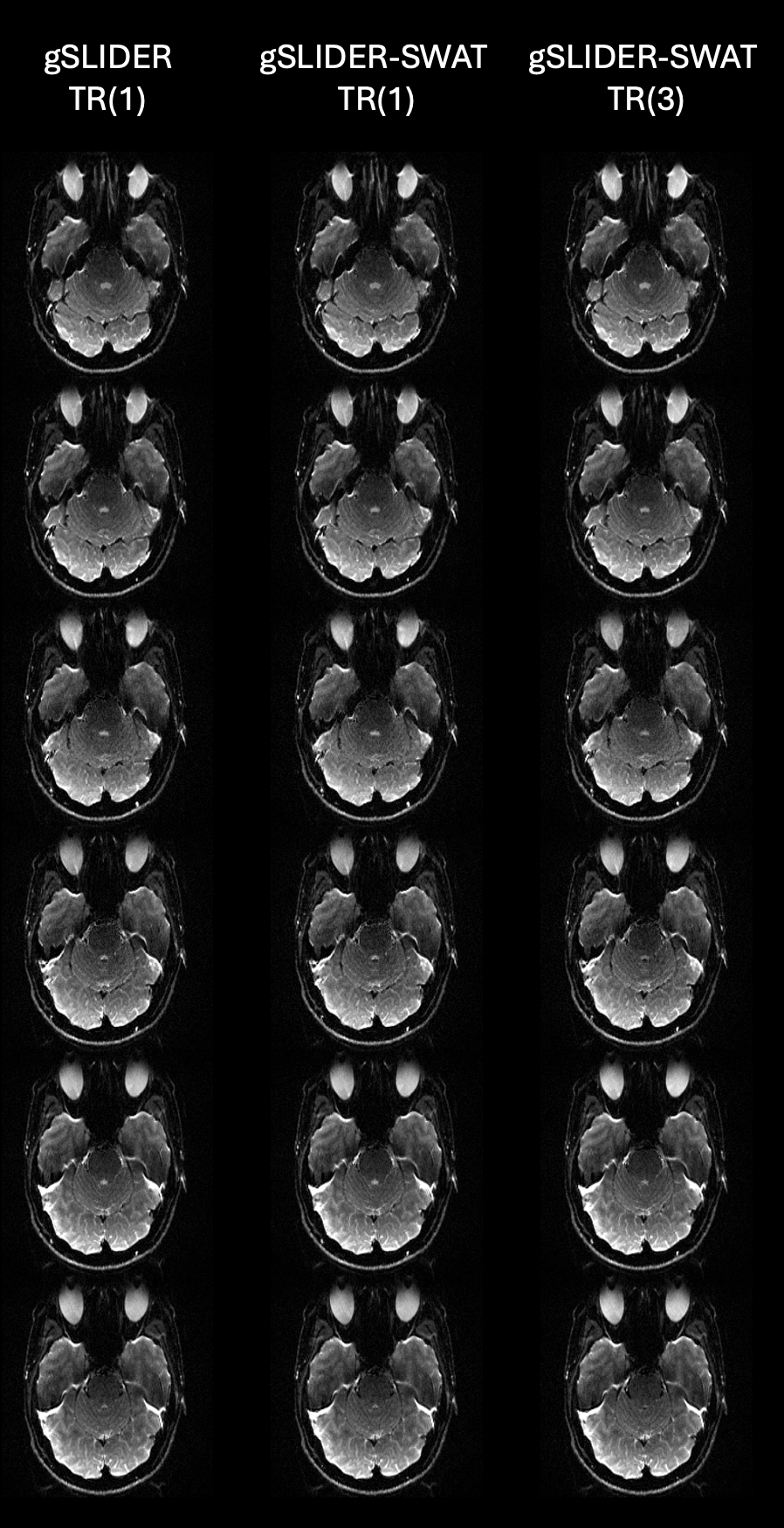

Supplement: Supplementary file 6 [file Image_6.jpeg]
